# Supplementary material for: Guiding cell adhesion and motility by modulating cross-linking and topographic properties of microgel arrays
Source: PLoS One. 2021 Sep 23;16(9):e0257495. doi: 10.1371/journal.pone.0257495 (PMC8460069; doi:10.1371/journal.pone.0257495)
Supplement: S1 Appendix — (DOCX) [file pone.0257495.s001.docx]

**Supplementary information for**

**Guiding cell adhesion and motility by modulating crosslinking and topographic properties of microgel arrays**

Janine Riegert^1¶^, Alexander Töpel^2,3¶^, Jana Schieren^1^, Renee Coryn^1^, Stella Dibenedetto^1^, Dominik Braunmiller^2,3^, Kamil Zajt^1^, Carmen Schalla^1^, Stephan Rütten^4^, Martin Zenke^1^, Andrij Pich^2,3^ and Antonio Sechi^1*^

^1^Institute of Biomedical Engineering, Dept. of Cell Biology, RWTH Aachen University, Pauwelsstrasse, 30, D-52074 Aachen, Germany

^2^Functional and Interactive Polymers, Institute of Technical and Macromolecular Chemistry, RWTH Aachen University, Worringerweg 2, D-52074, Aachen, Germany

^3^DWI, Leibniz Institute for Interactive Materials e.V.,

Forckenbeckstrasse, 50, D-52074 Aachen, Germany

^4^Electron Microscopy Facility, Institute of Pathology, RWTH Aachen University,

Pauwelsstrasse, 30, D-52074 Aachen, Germany

*: these authors equally contributed to this study

*Corresponding author: Antonio Sechi

Email: antonio.sechi@rwth-aachen.de

Telephone: 0049-241-8085248

# Materials and Methods

# Characteristics of PDMS used stamps

As shown in previous studies, the wavelength of PDMS stamps depends on the thickness of the oxide layer (directly related to both plasma activation time and plasma energy [1, 2]) and stretching of the PDMS stamps (S3 Figure).

# Additional information on microgels

The microgels exhibit a temperature responsive behavior, which was not affected by the use of surfactant during the synthesis (S1 Figure A). The volume phase transition temperature (VPTT) was also not affected by the surfactant (Table 1) being around 32°C, a value that has already been reported in previous studies.[3, 4] As shown in Suppl Figure 1B, the degree of microgel swelling (R_x_), calculated from the DLS data presented in Suppl Figure 1A, was almost unaffected by CTAB. Smaller microgels tend to have a more homogeneous structure within the polymer network, which can lead to a higher degree of microgel swelling.[5] A simple and effective strategy to influence the degree of microgel swelling (i.e., the stiffness) consists of changing cross-linker concentration.[6] As expected, microgel swelling was reduced at the higher cross-linker concentration (5mol%), an effect that can be explained by the reduced ability of these microgels to absorb water.

The contact area of the microgel, measured by AFM, was 1034 nm for microgels with 0wt% CTAB and 2.35mol%BIS (MG small) and decreased to 569 nm (MG medium) and 203 nm (MG small) with increasing amounts of CTAB. The height decreased from 46 nm to 4 nm. The low height of the microgels MG small lead to a high standard deviation (S2 Table). The highly cross linked microgel with a BIS content of 5mol% (MG large-stiff) were the tallest microgels, with a height of 94 nm. In contrast, the contact area decreased to 708 nm. The deformation values index (deformation=D_AFM_/h_AFM_) of microgels MG large and MG medium was in the range 22-25, while for the smallest microgels, due to the high standard deviation, this index was 59. The microgel MG large-stiff had a deformation index of 7.5, which was lower than that of the MG large. This followed our anticipated results of reduced deformation with increased cross-linker content.

# Array Characteristics

To prove the stability of microgels in aqueous media, the printed microgel arrays were incubated for 48 h in water. To compare how the morphology changed, AFM images of the samples were made before and after the incubation (Fig. 2 and S3 Fig.). From these images the height profiles could be determined. Additionally, the height profiles were used to determine the spacing and the height of the arrays on the surface in dry state. All microgels formed a homogeneous array structure after printing and after incubation in water, which proved that they were stable in water and maintained their directionality and dimensions. The target wavelength was in good agreement with the final array spacing (see S6 Table). The microgels arrays were taller after the printing process (dry conditions) when compared with the incubated structures. In the printing process, the microgel lines dried within the mold, leading to a deformed microgel structure. After swelling of the argon cross linked microgel arrays in water, the structure of the microgels recovered, leading to lines with reduced height, but increased diameter. The line structure was maintained by the argon plasma treatment, which led to crosslinking between the microgels.


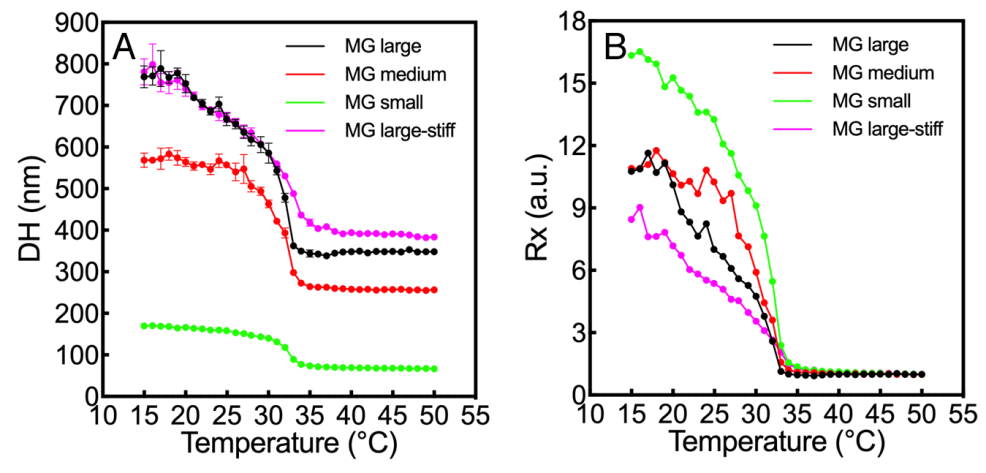


**S1 Figure. Analysis of temperature responsive properties of the microgels.** Temperature-dependent behavior of hydrodynamic diameter (A) and the degree of swelling (B). Note that the use of CTAB during the synthesis reduced microgel diameter in a concentration-dependent manner (A) but did not grossly affect microgel response to variations of temperature. Error bars in A indicate one standard deviation above and below the mean.


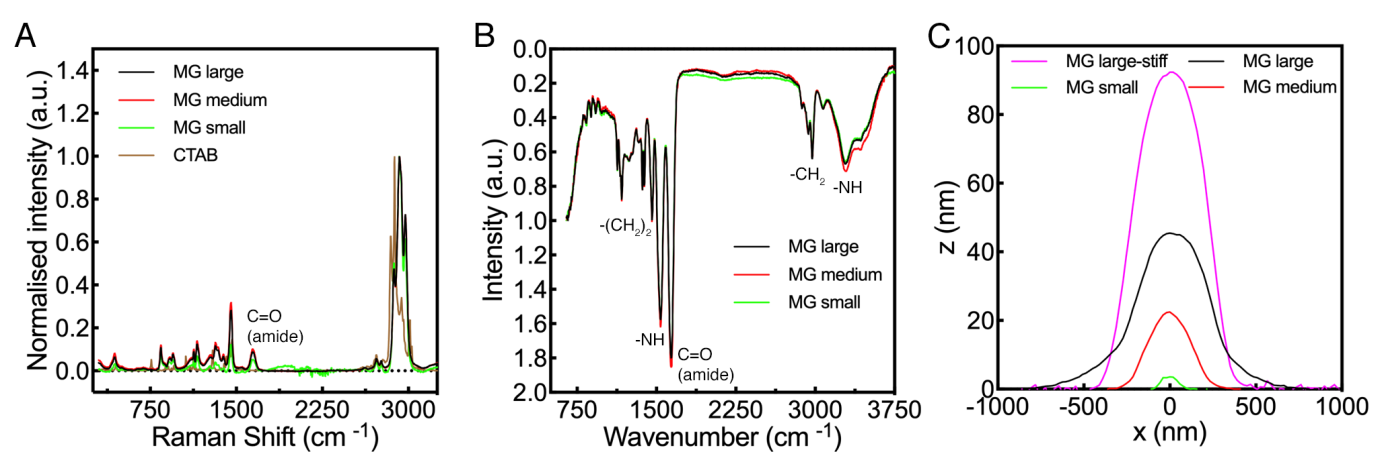


**S2 Figure. Analysis of microgel chemical composition and diameter in the presence or absence of CTAB.** (A-B) Raman and FTIR spectra showing that microgel synthesized in the absence or presence of CTAB have the same chemical composition. All microgels show the same bands in the Raman and FTIR spectra. In the Raman spectra, the carbonyl stretching bond (C=O) is at a shift of 1641 cm^-1^. Below a shift of 1500 cm^-1^, in the finger print area, the spectra of all samples overlap. In the FTIR spectra, the bands of the carbonyl stretching (C=O) at 1640 cm^-1^ and the amine band (-NH) at 1539 cm^-1^ overlap for all microgels. Note that at 1296 cm^-1^ the δCH_3_ rocking shift is observable in the Raman spectra, which correlates to CTAB. This shift is only visible in the pure CTAB, but not in the polymer spectra. (C) The height and contact area decrease with increasing amounts of CTAB during the synthesis. The contact area of the microgel, measured by AFM, is at 1034 nm for microgels with 0wt% CTAB and 2.35mol%BIS and decreases to 569 nm and 203 nm with increasing amounts of CTAB. The height decreases from 46 nm to 4 nm. The highly cross linked microgels, with a BIS content of 5mol%, are the tallest microgels achieving a height of 94 nm. In contrast, the contact area decreases to 708 nm.


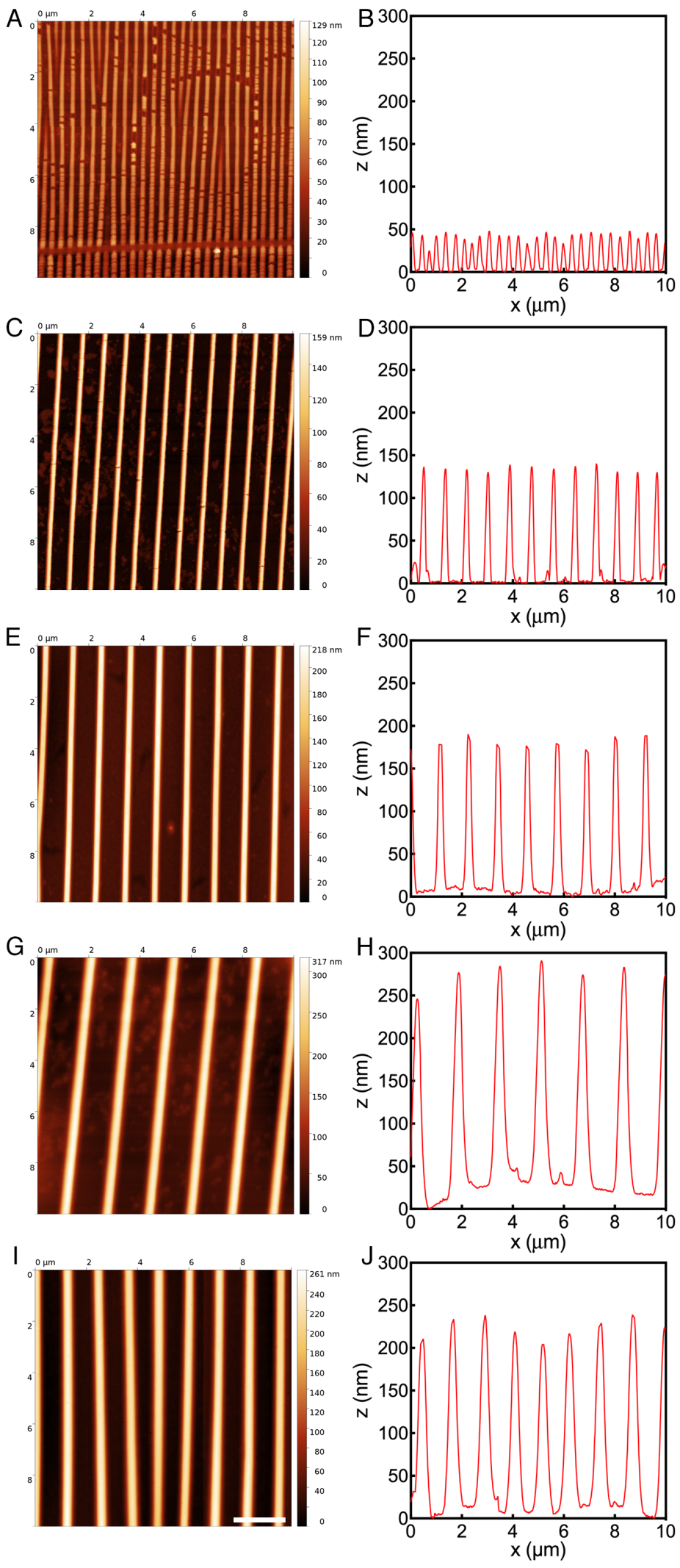


**S3 Figure. Atomic force microscopy analysis of dry microgel arrays.** Representative atomic force microscopy images and their plot profiles of microgel arrays printed on glass coverslips at a spacing of 300 (A, B), 800 (C, D), 1200 (E, F) and 1600 (G, H) nm and 1200 nm made by MG large-stiff (I, J). The legend on the side of each AFM image indicates the height of the arrays. Scale bar: 2 μm.


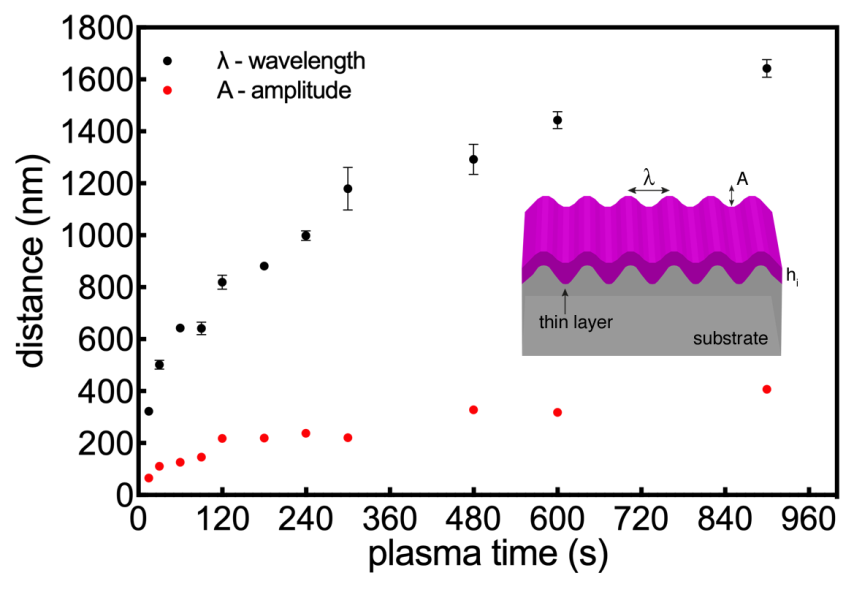


**S4 Figure. Effect of plasma time on the wavelength and amplitude of PDMS stamps used for microgel array printing.** Both stretching (133% of original size of PDMS stamp) and energy (100 W) were kept constant, leading to a dependence of the wavelength on the plasma activation time. At short activation times, the changes of wavelength have a larger magnitude than at longer plasma activation times. A similar behavior can be observed for the amplitude. The lower and upper technical limits for the wavelength of stamps are: 300 nm (15 sec activation time) and 1600 nm (900 sec activation time), respectively.


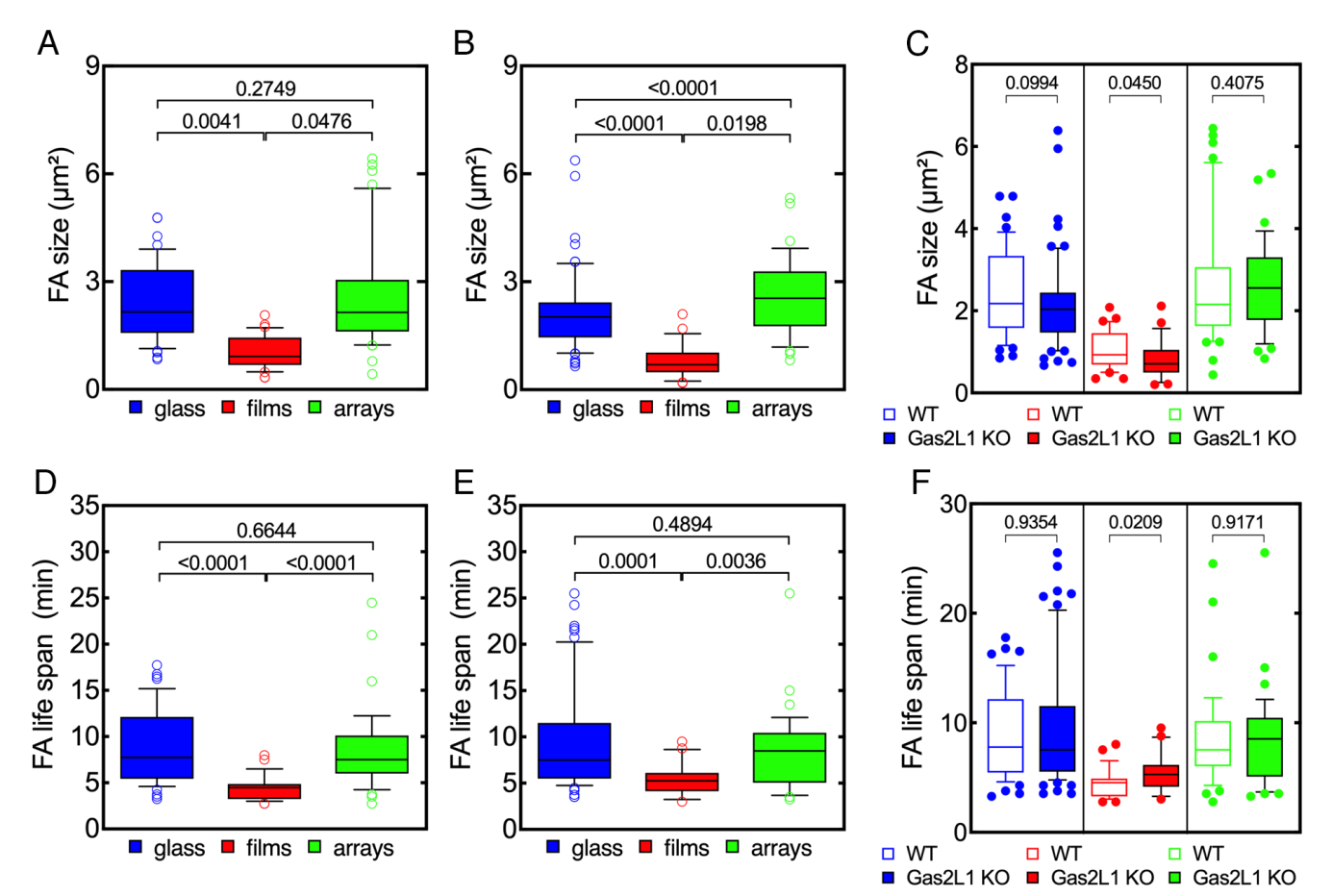


**S5 Figure. Impact of microgels on focal adhesion size and life span in wild-type and Gas2L1 KO Sertoli cells.** (A-C) Control and Gas2L1 KO cells show comparable focal adhesion sizes on microgel films and arrays. In both control and Gas2L1 KO cells, focal adhesion size is smaller in cells seeded on microgel films (A, B). Pairwise comparisons show a small, but significant, reduction of focal adhesion size in Gas2L1 KO cells that were seeded on to microgel films (C). (D-F) Control and Gas2L1 KO cells show comparable focal adhesion life span on glass and microgel films. Both control and Gas2L1 cells are characterized by smaller focal adhesion life span on microgel films compared to glass and microgel arrays substrates (D, E). Pairwise comparisons show comparable focal adhesion life spans in control and Gas2L1 KO cells on glass and microgel arrays, whereas focal adhesion life span is significantly increased in Gas2L1 KO cells on microgel films (F). Color code for C and F. Blue: glass; red: films; green: arrays. Numbers indicate p values.


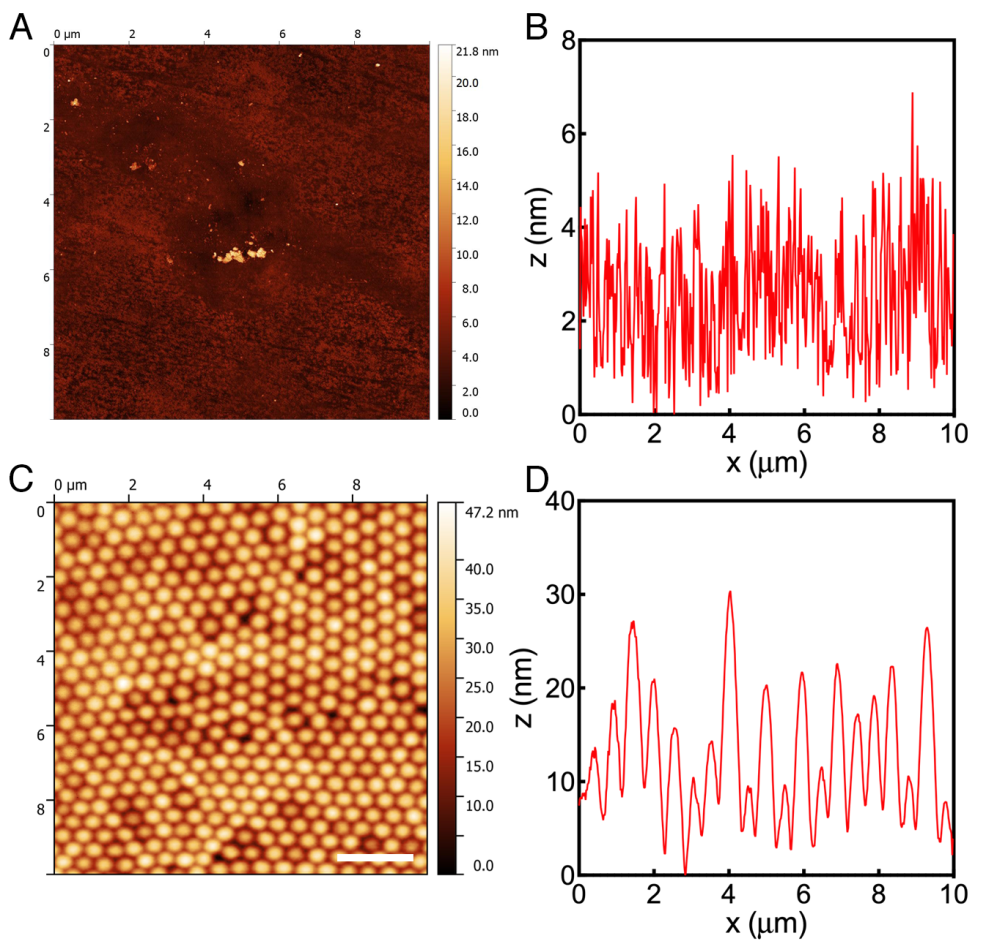


**S6 Figure. Atomic force microscopy analysis of bare glass and microgel films.** (A, B) refer to bare glass substrate, whereas (C, D) refer to a glass substrate coated with a homogeneous layer of MG large. The bare glass surface has a surface roughness of 5 nm, which means that it is relatively smooth compared to the microgels. The layer of microgel has a surface roughness of approximately 20-30 nm. The bare microgel has a height in AFM of 46 nm, indicating that the surface consists of densely packed microgels. Scale bar: 2 μm.

**S1 Table. Amounts of the chemical used for the preparation of the microgels.**

|  | **NIPAm** | **BIS** | | **AMPA** | | **CTAB** | | Concentration |
| --- | --- | --- | --- | --- | --- | --- | --- | --- |
|  | (g) | (g) | (mol%) | (g) | (mol%) | (g) | (wt%) | [mg mL^-1^] |
| MG large-stiff | 1.876 | 0.128 | 5.0 | 0.050 | 1.11 | 0 | 0 | 14.78 |
| MG large | 1.868 | 0.060 | 2.35 | 0.050 | 1.11 | 0 | 0 | 14.12 |
| MG medium | 1.877 | 0.603 | 2.35 | 0.050 | 1.11 | 0.005 | 0.5 | 12.53 |
| MG small | 1.877 | 0.060 | 2.35 | 0.050 | 1.11 | 0.045 | 2.5 | 15.87 |

NIPAm: *N*-isopropylacrylamide; BIS: *N,N*’-methylene bisacrylamide; AMPA: 2,2’-azobis-2- methylpropionamidine dihydrochloride; CTAB: cetyltrimethylammonium bromide

**S2 Table. Atomic force microscopy analysis of microgels.**

|  | **D_AFM_**  (nm) | **h_AFM_**  (nm) | **D_AFM_/h_AFM_**  (a.u.) |
| --- | --- | --- | --- |
| MG large-stiff | 708±36 | 94±6 | 7.5±0.3 |
| MG large | 1034±83 | 46±1 | 22.6±1.5 |
| MG medium | 569±99 | 23±2 | 25.1±3.3 |
| MG small | 203±54 | 4±1 | 59.4±21.4 |

D_AFM_: microgel particle diameter; h_AFM_: microgel particle height

**S3 Table. Average speed of Sertoli cells.**

| **Glass** |  | **Average speed (μm min^-1^)** | **S.D.** |
| --- | --- | --- | --- |
|  | WT | 0.304 | 0.1424 |
|  | Gas2L1 KO | 0.561 | 0.2804 |
| **Arrays** |  | **Average speed (μm min^-1^)** | **S.D.** |
|  | WT | 0.394 | 0.2344 |
|  | Gas2L1 KO | 0.943 | 0.6318 |
| **Films** |  | **Average speed (μm min^-1^)** | **S.D.** |
|  | WT | 0.185 | 0.1071 |
|  | Gas2L1 KO | 0.415 | 0.2727 |

**S4 Table. Parameters of focal adhesion turnover.**

| **Glass** |  | **FA assembly rate (μm^2^ sec^-1^)** | **S.D.** |
| --- | --- | --- | --- |
|  | WT | 0.003375 | 0.001318 |
|  | Gas2L1 KO | 0.004401 | 0.001526 |
| **Arrays** |  | **FA assembly rate (μm^2^ sec^-1^)** | **S.D.** |
|  | WT | 0.002695 | 0.0008895 |
|  | Gas2L1 KO | 0.003333 | 0.002357 |
| **Films** |  | **FA assembly rate (μm^2^ sec^-1^)** | **S.D.** |
|  | WT | 0.002236 | 0.0006499 |
|  | Gas2L1 KO | 0.00178 | 0.001073 |

| **Glass** |  | **FA disassembly rate (μm^2^ sec^-1^)** | **S.D.** |
| --- | --- | --- | --- |
|  | WT | 0.00309 | 0.001369 |
|  | Gas2L1 KO | 0.005493 | 0.004074 |
| **Arrays** |  | **FA disassembly rate (μm^2^ sec^-1^)** | **S.D.** |
|  | WT | 0.002479 | 0.001015 |
|  | Gas2L1 KO | 0.00334 | 0.001252 |
| **Films** |  | **FA disassembly rate (μm^2^ sec^-1^)** | **S.D.** |
|  | WT | 0.001703 | 0.001035 |
|  | Gas2L1 KO | 0.001893 | 0.00134 |

| **Glass** |  | **FA speed (nm sec^-1^)** | **S.D.** |
| --- | --- | --- | --- |
|  | WT | 130 | 55.74 |
|  | Gas2L1 KO | 191.9 | 65.51 |
| **Arrays** |  | **FA speed rate (nm sec^-1^)** | **S.D.** |
|  | WT | 115 | 47.25 |
|  | Gas2L1 KO | 132 | 67.84 |
| **Films** |  | **FA speed rate (nm sec^-1^)** | **S.D.** |
|  | WT | 94.79 | 46.85 |
|  | Gas2L1 KO | 92.04 | 46.63 |

| **Glass** |  | **FA size (μm^2^)** | **S.D.** |
| --- | --- | --- | --- |
|  | WT | 2.41 | 1.07 |
|  | Gas2L1 KO | 2.12 | 1.07 |
| **Arrays** |  | **FA size (μm^2^)** | **S.D.** |
|  | WT | 2.56 | 1.46 |
|  | Gas2L1 KO | 2.59 | 1.09 |
| **Films** |  | **FA size (μm^2^)** | **S.D.** |
|  | WT | 1.05 | 0.46 |
|  | Gas2L1 KO | 0.80 | 0.46 |

| **Glass** |  | **FA life span (min)** | **S.D.** |
| --- | --- | --- | --- |
|  | WT | 8.91 | 4.07 |
|  | Gas2L1 KO | 9.59 | 5.55 |
| **Arrays** |  | **FA life span (min)** | **S.D.** |
|  | WT | 8.47 | 4.31 |
|  | Gas2L1 KO | 8.31 | 4.23 |
| **Films** |  | **FA life span (min)** | **S.D.** |
|  | WT | 4.42 | 1.41 |
|  | Gas2L1 KO | 5.51 | 1.85 |

**S5 Table. Zyxin turnover at focal adhesions.**

| **Glass** |  | **Mobile fraction (a.u.)** | **S.D.** |
| --- | --- | --- | --- |
|  | WT | 0.46 | 0.086 |
|  | Gas2L1 KO | 0.36 | 0.12 |
| **Arrays** |  | **Mobile fraction (a.u.)** | **S.D.** |
|  | WT | 0.38 | 0.12 |
|  | Gas2L1 KO | 0.52 | 0.16 |
| **Films** |  | **Mobile fraction (a.u.)** | **S.D.** |
|  | WT | 0.49 | 0.11 |
|  | Gas2L1 KO | 0.39 | 0.09 |

**S6 Table. Microgel array features.**

|  | **Plasma time**  (sec) | **Theoretical wavelength**  (nm) | **Spacing after printing**  (nm) | **Height after printing**  (nm) | **Spacing after equilibration**  (nm) | **Height after equilibration**  (nm) |
| --- | --- | --- | --- | --- | --- | --- |
| MG large-stiff | 480 | 1200 | 1248±1 | 222±3 | 1274±20 | 93±7 |
| MG large | 900 | 1600 | 1618±5 | 259±7 | 1548±65 | 134±11 |
| MG large | 480 | 1200 | 1208±37 | 191±14 | 1125±14 | 57±5 |
| MG medium | 120 | 800 | 843±5 | 126±20 | 839±30 | 44±2 |
| MG small | 15 | 300 | 371±7 | 47±3 | 365±8 | 15±1 |

**S7 Table A. Statistical analysis of B16F1 average speed (for data in Fig. 9).**

| **Multiple comparisons** | **Significant?** | **Summary** | **p value** |
| --- | --- | --- | --- |
| 300 nm vs. 800 nm | Yes | **** | <0.0001 |
| 300 nm vs. 1200 nm | Yes | **** | <0.0001 |
| 300 nm vs. 1600 nm | Yes | **** | <0.0001 |
| 300 nm vs. films | Yes | **** | <0.0001 |
| 300 nm vs. glass | Yes | **** | <0.0001 |
| 800 nm vs. 1200 nm | Yes | *** | 0.0002 |
| 800 nm vs. 1600 nm | Yes | **** | <0.0001 |
| 800 nm vs. films | Yes | **** | <0.0001 |
| 800 nm vs. glass | Yes | **** | <0.0001 |
| 1200 nm vs. 1600 nm | Yes | **** | <0.0001 |
| 1200 nm vs. films | Yes | **** | <0.0001 |
| 1200 nm vs. glass | Yes | **** | <0.0001 |
| 1600 nm vs. films | Yes | ** | 0.0086 |
| 1600 nm vs. glass | Yes | **** | <0.0001 |
| Films vs. glass | Yes | **** | <0.0001 |

**S7 Table B. Statistical analysis of B16F1 directionality (for data in Fig. 9).**

| **Multiple comparisons** | **Significant?** | **Summary** | **p value** |
| --- | --- | --- | --- |
| 300 nm vs. 800 nm | Yes | **** | <0.0001 |
| 300 nm vs. 1200 nm | Yes | **** | <0.0001 |
| 300 nm vs. 1600 nm | Yes | **** | <0.0001 |
| 300 nm vs. films | Yes | **** | <0.0001 |
| 300 nm vs. glass | Yes | **** | <0.0001 |
| 800 nm vs. 1200 nm | No | ns | >0.9999 |
| 800 nm vs. 1600 nm | No | ns | 0.9997 |
| 800 nm vs. films | Yes | **** | <0.0001 |
| 800 nm vs. glass | Yes | **** | <0.0001 |
| 1200 nm vs. 1600 nm | No | ns | >0.9999 |
| 1200 nm vs. films | Yes | **** | <0.0001 |
| 1200 nm vs. glass | Yes | **** | <0.0001 |
| 1600 nm vs. films | Yes | **** | <0.0001 |
| 1600 nm vs. glass | Yes | **** | <0.0001 |
| Films vs. glass | No | ns | 0.3405 |

**S8 Table A. Statistical analysis of focal adhesion assembly rate (for data in Fig. 9).**

| **Multiple comparisons** | **Significant?** | **Summary** | **p value** |
| --- | --- | --- | --- |
| 300 nm vs. 800 nm | Yes | **** | <0.0001 |
| 300 nm vs. 1200 nm | No | ns | 0.7364 |
| 300 nm vs. 1600 nm | Yes | **** | <0.0001 |
| 300 nm vs. films | Yes | **** | <0.0001 |
| 300 nm vs. glass | No | ns | 0.5574 |
| 800 nm vs. 1200 nm | Yes | *** | 0.0001 |
| 800 nm vs. 1600 nm | No | ns | 0.3029 |
| 800 nm vs. films | No | ns | 0.7385 |
| 800 nm vs. glass | Yes | **** | <0.0001 |
| 1200 nm vs. 1600 nm | No | ns | 0.0811 |
| 1200 nm vs. films | Yes | **** | <0.0001 |
| 1200 nm vs. glass | No | ns | 0.0655 |
| 1600 nm vs. films | Yes | * | 0.0187 |
| 1600 nm vs. glass | Yes | **** | <0.0001 |
| Films vs. glass | Yes | **** | <0.0001 |

**S8 Table B. Statistical analysis of focal adhesion disassembly rate (for data in Fig. 9).**

| **Multiple comparisons** | **Significant?** | **Summary** | **p value** |
| --- | --- | --- | --- |
| 300 nm vs. 800 nm | Yes | * | 0.0193 |
| 300 nm vs. 1200 nm | Yes | * | 0.0489 |
| 300 nm vs. 1600 nm | No | ns | 0.1663 |
| 300 nm vs. films | No | ns | 0.9831 |
| 300 nm vs. glass | Yes | **** | <0.0001 |
| 800 nm vs. 1200 nm | Yes | **** | <0.0001 |
| 800 nm vs. 1600 nm | No | ns | 0.9792 |
| 800 nm vs. films | Yes | ** | 0.0036 |
| 800 nm vs. glass | Yes | **** | <0.0001 |
| 1200 nm vs. 1600 nm | Yes | **** | <0.0001 |
| 1200 nm vs. films | No | ns | 0.3062 |
| 1200 nm vs. glass | Yes | **** | <0.0001 |
| 1600 nm vs. films | Yes | * | 0.0434 |
| 1600 nm vs. glass | Yes | **** | <0.0001 |
| Films vs. glass | Yes | **** | <0.0001 |

# Supplementary references

1. Brüx R, Hiltl S, Schröder V, von Essen C, Böker A. Designing Zwitterionic SiO2NH2-Au Particles with Tunable Patchiness using Wrinkles. Particle & Particle Systems Characterization. 2014;31(8):871-8. doi: 10.1002/ppsc.201300354.

2. Genzer J, Groenewold J. Soft matter with hard skin: From skin wrinkles to templating and material characterization. Soft Matter. 2006;2(4):310-23. doi: 10.1039/B516741H.

3. Heskins M, Guillet JE. Solution Properties of Poly(N-isopropylacrylamide). Journal of Macromolecular Science: Part A - Chemistry. 1968;2(8):1441-55. doi: papers3://publication/doi/10.1080/10601326808051910.

4. Saunders BR, Laajam N, Daly E, Teow S, Hu X, Stepto R. Microgels: From responsive polymer colloids to biomaterials. Advances in colloid and interface science. 2009;147-148:251-62. doi: 10.1016/j.cis.2008.08.008. PubMed PMID: 18809173.

5. Arleth L, Xia X, Hjelm RP, Wu J, Hu Z. Volume transition and internal structures of small poly( N-isopropylacrylamide) microgels. J Polym Sci B Polym Phys. 2005;43(7):849-60. doi: papers3://publication/doi/10.1002/polb.20375.

6. Mourran A, Wu Y, Gumerov RA, Rudov AA, Potemkin II, Pich A, et al. When Colloidal Particles Become Polymer Coils. Langmuir. 2016;32(3):723-30. doi: papers3://publication/doi/10.1021/acs.langmuir.5b03931.
