# Supplementary material for: Guiding cell adhesion and motility by modulating cross-linking and topographic properties of microgel arrays
Source: PLoS One. 2021 Sep 23;16(9):e0257495. doi: 10.1371/journal.pone.0257495 (PMC8460069; doi:10.1371/journal.pone.0257495)
Supplement: Attachment — Submitted filename: PONE-D-21-09835_comments.pdf [file pone.0257495.s003.pdf]

The authors have addressed some of the comments raised, but left some of the most important comments regarding the validation of some of their conclusions. Despite the replies made, the associated text and discussion were not corrected appropriately. I still disagree with these conclusions and do not recommend publication of this manuscript unless these aspects are fully and appropriately addressed.

5. In Fig 6. FA assembly seems to be differentially regulated by Gas2L1 expression when cultured on glass, but not when cultured on films or arrays. Trends (relative rates of assembly/disassembly of FAs) are overall very similar for WT and KO lines. This does not support the notion that Gas2L1 is mediating the sensing of the microgel properties. It is not clear what properties are particularly targeted either in this study (topography/size of microgels, chemistry, mechanics).

In this part of the study, we have not analysed specific microgel properties but the impact of microgels on FA dynamics in control cells and cells lacking Gas2L1. We clearly show that microgels are effective in regulating the motility and adhesion of Sertoli cells. Furthermore, FA assembly and disassembly rates are both reduced in Gas2L1 KO cells compared to control cells on microgels arrays and films (see suppl. Table S4) indicating that Gas2L1 is somehow involved in sensing of microgel substrata. Obviously, we do not know, at this stage, the molecular mechanisms for this process that will be investigated in future studies.

R: I am not disputing the fact that microgels regulate the directionality of cell motility. I am disputing the proposed role of Gas2L1 on this process. In Fig 5 (A-D), cells display high speed on the array compared to glass or film substrates, whether they are WT or KO, although the speed of KO cells is overall reduced. The trend in directionality is also the same. This is also mirrored by the identical trends observed in Fig 6 (assembly and disassembly rates, and FA speed, are reduced on films compared to arrays, both for WT and KO). Therefore, I conclude that, although Gas2L1 has an impact on cell adhesion and motility, it does not regulate the sensing of the topography. This needs to be corrected (and highlighted) in the text, abstract, intro, discussion and conclusion.

Interestingly, such effect is more prominent in Sertoli cells harboring a knockout of Gas2L1, a component of the cytoskeleton that mediates the interaction between microtubules and microfilaments. Moreover, on microgel arrays, the kinetics of the focal adhesion protein zyxin is decreased in wild-type and increased in Gas2L1 KO Sertoli cells. Finally, increasing microgel cross-linking causes a stronger reduction of focal adhesion turnover in Gas2L1 KO cells.

"we further reasoned that the present microgel system could be used to preferentially modulate the migration of wild-type or Gas2L1 KO Sertoli cells. A correct hypothesis would result in a clear difference in the rate of motility between the two Sertoli cell lines with the Gas2L1 cells migrating faster than wild-type cells."

P21. L466. Again, I am not disputing that the WT and KO migrate at different rate, they do. But they respond in a similar way to microgels and their topography, compared to glass. Therefore, the microgel system is not preferentially modulating the migration of WT or KO cells. Gas2L1 has an impact on migration, independent of these substrates.

"A closer analysis of the data showed that the ratio between the average speeds of Gas2L1 KO and wild-type cells was higher for cells on microgel films and microgel arrays than for cells on glass coverslips (S3 Table), clearly showing a more pronounced impact of microgels on the migration of Gas2L1 KO Sertoli cells."

P22, L493. " Conversely, in cells on microgel arrays, focal adhesion assembly was significantly reduced only in Gas2L1 KO cells (Fig. 6A-B, D-E)."

"It is important to note that the ratio between the assembly rate of Gas2L1 KO and wild-type cells was much smaller in cells on microgel films (S4 Table). The ratios for focal adhesion disassembly rate and speed followed a similar trend (S4 Table). Similar comparisons also showed that the focal adhesion size ratio was higher for cells on microgel arrays, whereas focal adhesion life span ratio was higher for cells on microgel films (S4 Table). Collectively, these findings demonstrate that the surface-grafted microgels can be used as an effective system to modulate focal adhesion dynamics in Sertoli cells and have a larger impact on Gas2L1 KO cells."

" Furthermore, the ratio between the mobile fractions of zyxin in wild-type and Gas2L1 KO cells was increased in cells seeded on microgel arrays (S5 Table). Thus, microgel arrays preferentially modulated zyxin kinetics in Gas2L1 KO cells, thus serving as an effective tool for highlighting differences of focal adhesion behavior between genotypically diverse cell types."

R: We noted the effort to characterise ratios between migration rates, directionality, rates of assembly/disassembly etc. between KO and WT and compare their ratios, but note that, as stated above, the overall trends remain unchanged and there is no indication that the ratios reported in Tables S3-S5 demonstrate quantitative differences in the response of KO and WT cells to patterns. Considering the standard deviations, the min/max ratios (calculated from minimising/maximising the corresponding values before calculating min/max ratios) are significantly overlapping.

Overall, the role of Gas2L1 on cell migration is clear, but its role on sensing topography of the microgels studied is not. This should be corrected in the manuscript throughout and clearly stated.

6. In Fig 7, the mobile fractions measured for the KO cells are lower than for WT, on glass. However, in Fig 6, the assembly and disassembly rates of FA are faster. The two observations seem to be contradicting.

The mobile fraction of zyxin reflects its dynamic behaviour within focal adhesions. Such behaviour may or may not reflect the dynamic behaviour of focal adhesions that depends on the function of several proteins. Hence, in our opinion, the more static behaviour of zyxin in KO cells on glass does not necessarily represent a "functional" contradiction. In fact, it is possible that the behaviour of one single focal adhesion protein is affected in a certain way, whereas focal adhesions as a whole behave in a opposite way.

R: This still needs to be stated and discussed. There is no evidence for the behaviour proposed by the authors. I do not recommend suggesting it without evidence or without reference to appropriate literature.

7. In Fig 9, trends are proposed for the cell response to different microgels. E.g. "their average speed and directionality were greatly reduced on 800, 1200 and 1600 nm arrays". Considering that there is no apparent statistical trend (the averages do not even follow a clear trend when the microgel size was varied from 300 to 1600 nm), any notion that the microgel size (and associated topography?) is regulating cell adhesion and motility should be removed from this manuscript. The only clear difference observed is between glass/film and arrays. But again, this breaks down when analysing the impact of substrate topography/patterning on FA assembly/disassembly.

We thank the reviewer for the interesting comment on our interpretation of the data. We based our interpretation of the data and statements on the statistical analysis (see suppl. Tables S7 and S8). In our opinion there is a clear trend showing that the average speed decreases as the microgel spacing increases.

R: I disagree. Fig 9 does not show stats, but 1600 nm is clearly above 1200 and 800 nm. Similarly, other processes quantified in Figure 9 clearly do not show trends. Some stats should be included directly in this figure, to facilitate its quantitative analysis.

Likewise, spacing greater than 300 nm consistently leads to higher directionality of cell motility. We cannot, at present, precisely explain why cells on 1600 nm microgel arrays regain part of their motility. However, we note that the 1600 nm microgel arrays form a pyramidal structure characterised by one microgel sitting on the top of two microgels. This technical limitation arises by both the fact that we have used the largest possible PDMS stamp to print the 1600 nm arrays, and that physical limitations do not allow to generate larger microgels during the synthesis. Regardless of these current technical drawbacks, it is certainly important to investigate how cell behaviour changes when the distance between adjacent arrays increases.

R: clearly this calls for a modification of the conclusions. The microgel size does not clearly modulate migration. Only 300 nm gels do.

10. Conclusions made in the discussion, specifically (but not only) "the migration of B16F1 cells is inversely correlated with microgel array spacing" are not supported by the data presented. This needs to be carefully rewritten. Similarly, "In this context, we have developed a (...) cell adhesion and migration". I do not see evidence that the system presented allow to modulate actin cytoskeleton architecture. There is no control in the phenotypes observed and no clear trends when correlating spacing and migration/FA dynamics are observed. The cytoskeleton architecture has not been systematically studied or characterised.

In our opinion, the statement "the migration of B16F1 cells is inversely correlated with microgel array spacing" is correct and supported by Fig. 9 (with the possible exception of the motility of cells on 1600 nm microgel arrays).

R: As noted above, there is no trend between migration speed, directionality and assembly/disassembly rates in Figure 9. According to Table S7 cells migrate faster on 1600

nm gels. The directionality is only significantly different for cells on 300 nm patterns. The assembly/disassembly rates follow a see-saw pattern, which does not constitute a trend.

11. The conclusion that "the variation of microgel array topographic and mechanical features can efficiently be used for the modulation of cell adhesion and motility" is misleading. There is no real control achieved (which would be evidenced by trends with microgel size for example). The only trends observed are the response of cells to films and arrays compared to glass. But this could be the result of differences in adhesive protein adsorption promoting differences in cell adhesion.

In our opinion, the statement "the variation of microgel array topographic and mechanical features can efficiently be used for the modulation of cell adhesion and motility" highlighted by the reviewer is supported by solid experimental evidence. As to the topography, we have not only compared cell motility and adhesion on glass to the same biological processes on films and arrays, but also films to arrays. Furthermore, we have changed arrays spacing (i.e., microgel topography) and clearly show that it can modulate cell adhesion and motility. Also in this case, the comparison was done with glass, but also with films and between pairs of different array spacings (see statistical analysis in the suppl. data). Regarding the mechanical feature (i.e., content of cross-linker), we clearly show that focal adhesion turnover is clearly modulated by this microgel feature in both WT and Gas2L1 KO cells.

R: As noted above, this still requires revising. This study presents some interesting results, but I dispute some of its conclusions and this should not be discarded.

12. Fig. 7. It would be useful to show some of the normalised fluorescence intensities prior to photobleaching in the traces presented.

As the reviewer certainly knows, the normalised intensities prior to photobleaching are equal to 1 and will be visualised as curves parallel to the X axis for all the conditions (i.e., glass, films and arrays). In our opinion, the information will not add any relevant detail to the figure but introducing it will cause the curves to be squeezed together (because the Y axis will include a range of values up to 1 or more) thus making any difference difficult to appreciate.

R: The value of presenting normalised fluorescence intensities directly prior to bleaching (for a few tens of s is to clearly show whether some gradual photobleaching of the systems was observed simply during imaging.

Statistical Analysis.
Experiments should all be carried out at least in triplicates, rather than duplicates and triplicates.
