# Supplementary material for: Guiding cell adhesion and motility by modulating cross-linking and topographic properties of microgel arrays
Source: PLoS One. 2021 Sep 23;16(9):e0257495. doi: 10.1371/journal.pone.0257495 (PMC8460069; doi:10.1371/journal.pone.0257495)
Supplement: Attachment — Submitted filename: Response to Reviewers.docx [file pone.0257495.s002.docx]

**Response to reviewers**

**Reviewer #1:**

*This manuscript describes the patterning of hydrogel microparticles and the response of cell spreading to the corresponding interfaces. The impact of the resulting patterns on the spreading and motility of Sertoli cells and Gas2L1 KO cells was characterised. The dynamics of focal adhesions was then studied. The authors then switch to the study of B16F1 cells for the study of the impact of microgel size on cell adhesion, motility and adhesion turnover. Finally, the impact of microgel mechanics was studied. Such phenomena and the impact of regulators of actin assembly on sensing of such topographies are interesting, but important aspects are not properly characterised or insufficiently detailed. The following should be addressed prior to considering this manuscript any further:*

*1. Understanding factors regulating the sensing of nanotopography is important, but it is not clear why select Gas2L1 specifically. Many other regulators of actin assembly or FA regulators could have been picked up.*

As correctly pointed out by the reviewer, several actin-associated proteins and regulators of FA dynamics are likely responsible for cellular sensing of nanotopography. We have recently started to characterise the function(s) of Gas2L1, which, so far, is poorly defined at molecular level. Because we have demonstrated that Gas2L1 can regulate focal adhesion dynamics and cell motility on classical glass and plastic substrata (see Gamper et al., 2016), in the present study we wanted to test whether this protein plays a role in the regulation of cell motility and focal adhesion dynamics on microgel arrays. Since we aim at developing our microgel substrata as tools for controlling cell motility and adhesion, future studies will certainly focus on characterising the molecular make up cells need to respond to various nano topographies.

*2. In addition, there are other platforms accessible to regulate nanoscale geometry and topography. It is not clear why this specific microgel platform was selected and how it allows to solve unanswered questions on the role of Gas2L1. Beyond the fact that Gas2L1 regulates motility, a fact previously established, this study does not demonstrate further roles for this player in topography sensing.*

Certainly, several platforms could be used to investigate the influence of nanoscale geometry and topography on cell motility and adhesion. As stated in the manuscript, our study was aimed at better understanding the impact of microgels on cell motility and adhesion thus complementing and expanding our previous study (Sechi et al., 2016). In addition, although the present study was not aimed at fully understanding the molecular basis of function of Gas2L1, it provides clear evidence that in the absence of this protein cells behave differently on microgels compared to controls (see Fig. 6 and 7). Future study will certainly focus on the detailed characterisation of Gas2L1 function in these processes.

*3. Similarly, it is not clear why the two cell models selected are particularly appropriate to study the impact of microgel pattern topographies on cell adhesion. It is also odd that some of the experiments are carried out only with B16F1 cells when most of the study focuses on Sertoli cells. If the aim of the study is to mimic topographical features present in the matrix, why not carry out all experiments in the same cell type. The argument presented, that clearer trends could be observed breaks down since no real trend correlating motility or FA turnover with microgel topography (and size) is observed. Given that all other experiments were carried out with Sertoli cells, studying the impact of microgel size on motility and FA turnover seems important.*

Our study could be considered as divided into two parts. In the first one, we wanted to answer two questions: 1) Do, and to which extent, surface-grafted microgel arrays influence the motility and adhesion of Sertoli cells? 2) Has the absence of Gas2L1 expression in these cells any effect on their motile and adhesive properties? Needless to say, more must be done to fully characterise the impact of microgels (in their various configurations) on Sertoli cells, however our study provides already some interesting findings. In the second part, as stated in the manuscript, we have decided to switch to B16F1 cells in order to be able to compare the influence of microgel arrays with different spacing to our previous study (Sechi et al., 2016), which focussed exclusively on B16F1 cells. Certainly, future studies will aim at fully characterising such aspects in more detail.

*4. The differences observed in terms of cell motility and FA turnover are proposed to result from differences in matrix. Clearly cells sense and align to the patterns generated, but this was previously reported. However, differences observed between glass, films and arrays (patterns) could arise from differences in cell adhesive protein adsorption. I could not find details of any specific protein adsorption or biofunctionalisation with peptides, therefore I assume that this was not controlled. This aspect should be investigated for the different substrates studied (including differences in microgel diameters and stiffness).*

We thank you the reviewer for touching this important issue. In the present study, we did not coat the microgels with proteins or peptides since we did not aim at analysing the effect of protein absorption. We cannot, at present, rule completely out that different protein absorption on glass, films and arrays may in part contribute to the effect observed. It is certainly an important aspect of this work that we will investigate in future studies.

*5. In Fig 6. FA assembly seems to be differentially regulated by Gas2L1 expression when cultured on glass, but not when cultured on films or arrays. Trends (relative rates of assembly/disassembly of FAs) are overall very similar for WT and KO lines. This does not support the notion that Gas2L1 is mediating the sensing of the microgel properties. It is not clear what properties are particularly targeted either in this study (topography/size of microgels, chemistry, mechanics).*

In this part of the study, we have not analysed specific microgel properties but the impact of microgels on FA dynamics in control cells and cells lacking Gas2L1. We clearly show that microgels are effective in regulating the motility and adhesion of Sertoli cells. Furthermore, FA assembly and disassembly rates are both reduced in Gas2L1 KO cells compared to control cells on microgels arrays and films (see suppl. Table S4) indicating that Gas2L1 is somehow involved in sensing of microgel substrata. Obviously, we do not know, at this stage, the molecular mechanisms for this process that will be investigated in future studies.

*6. In Fig 7, the mobile fractions measured for the KO cells are lower than for WT, on glass. However, in Fig 6, the assembly and disassembly rates of FA are faster. The two observations seem to be contradicting.*

The mobile fraction of zyxin reflects its dynamic behaviour within focal adhesions. Such behaviour may or may not reflect the dynamic behaviour of focal adhesions that depends on the function of several proteins. Hence, in our opinion, the more static behaviour of zyxin in KO cells on glass does not necessarily represent a “functional” contradiction. In fact, it is possible that the behaviour of one single focal adhesion protein is affected in a certain way, whereas focal adhesions as a whole behave in a opposite way. Tackling the giant task of analysing the impact of microgels on the 100+ known focal adhesion proteins will certainly help to clarify this apparent inconsistency.

*7. In Fig 9, trends are proposed for the cell response to different microgels. E.g. "their average speed and directionality were greatly reduced on 800, 1200 and 1600 nm arrays". Considering that there is no apparent statistical trend (the averages do not even follow a clear trend when the microgel size was varied from 300 to 1600 nm), any notion that the microgel size (and associated topography?) is regulating cell adhesion and motility should be removed from this manuscript. The only clear difference observed is between glass/film and arrays. But again, this breaks down when analysing the impact of substrate topography/patterning on FA assembly/disassembly.*

We thank the reviewer for the interesting comment on our interpretation of the data. We based our interpretation of the data and statements on the statistical analysis (see suppl. Tables S7 and S8). In our opinion there is a clear trend showing that the average speed decreases as the microgel spacing increases. Likewise, spacing greater than 300 nm consistently leads to higher directionality of cell motility. We cannot, at present, precisely explain why cells on 1600 nm microgel arrays regain part of their motility. However, we note that the 1600 nm microgel arrays form a pyramidal structure characterised by one microgel sitting on the top of two microgels. This technical limitation arises by both the fact that we have used the largest possible PDMS stamp to print the 1600 nm arrays, and that physical limitations do not allow to generate larger microgels during the synthesis. Regardless of these current technical drawbacks, it is certainly important to investigate how cell behaviour changes when the distance between adjacent arrays increases.

*8. No statistical analysis results are reported in Fig 9.*

We thank you the reviewer for highlighting this unclear point in the manuscript. The statistical analysis is provided in the supplementary Tables S7 and S8, indeed. Given the multiple statistical comparisons, we have preferred to provide the analysis in Tables to avoid a very crowded and confusing figure. We now specify in the Tables S7 and S8 that the statistical analysis refers to Figure 9.

*9. What is the actual stiffness of the microgels used in Fig 10? This should be measured.*

We thank the reviewer for pointing out this issue and are happy to address his question. This is one of the interesting questions in microgel research, but yet an unanswered one. With classical rheological methods, it is not possible to measure the mechanical properties of a single microgel. Those methods only deliver data on the properties of the colloidal suspension. A promising technique to determine the mechanical properties of a single microgel is atomic force microscopy. In several articles, attempts were made to measure the mechanical properties, but yet a standard model is missing. Furthermore, the results must be judged carefully as the basic problem is the assumption that microgels are fully elastic macroscopic continua, while they have a heterogeneous crosslinked structure. A good discussion on this topic is made by F. Schulte (Microgels at solid/fluid interfaces investigated by atomic force microscopy. (RWTH Aachen University, 2020). doi:10.18154/RWTH-2020-10326 Chapter 8 (p94 f)) and we can recommend it for further information. It is also important to note that all current state-of-the-art methods are based on assumptions to determine microgel stiffness. A theory about predicting the shear modulus of microgels from the information of size and cross-linking density has been demonstrated for PNIPAm-based microgels (Hashmi, S. M. & Dufresne, E. R. Mechanical properties of individual microgel particles through the deswelling transition. Soft Matter 5, 3682-3688 (2009)). In this study, the microgels shear modulus is defined as:

$$G=\left( \frac{\phi_{0}{\cdot k}_{B}\cdot T}{2\cdot N_{gel}{\cdot\alpha}^{3}} \right)\left( \frac{\phi}{\phi_{0}} \right)^{1/3}$$

(eq1)

| $G$ | the particle shear modulus |
| --- | --- |
| $\phi$ | volume fraction of the particle in swollen and collapsed state (0) |
| $k_{B}$ | Boltzmann constant, |
| $T$ | Temperature |
| $N_{gel}$ | the average number of monomers between cross-links |
| $\alpha$ | the monomer size |

The volume fraction of a microgel is defined as:

$$\phi=\frac{V_{polymer}}{V_{particle}}$$

(eq4)

The equation can be reshaped to:

$$\left( \frac{\phi}{\phi_{0}} \right)^{1/3}=\left( \frac{\frac{V_{polymer}}{V_{particle}}}{\frac{V_{polymer}}{V_{collapsed}}} \right)^{1/3}=\frac{R_{h,50}}{R_{h}}$$

(eq5)

The value of $N_{gel}\alpha^{3}$ is the average volume of the segment between cross-links, which is equal to $\frac{V_{polymer}}{2N_{c}}$, as one crosslinker connects two sub-chains.

$$\frac{\phi_{0}}{2\cdot N_{gel}\cdot\alpha^{3}}=\frac{\frac{V_{polymer}}{V_{0}}}{2N_{gel}\alpha^{3}}=\frac{N_{c}}{V_{0}}$$

(eq6)

| $\phi_{0}$ | volume fraction of the particle in collapsed state |
| --- | --- |
| $V_{polymer}$ | volume of the polymer |
| $V_{0}$ | volume of the microgel in the collapsed state |
| $N_{c}$ | the total number of cross-links in the particle |
| $N_{gel}$ | the average number of monomers between cross-links |
| $\alpha$ | the monomer size |

$$G=\left( \frac{N_{c}{\cdot k}_{B}\cdot T}{V_{0}} \right)\frac{R_{h,50}}{R_{h}}=\left( \frac{N_{c}\cdot k_{B}\cdot T}{\frac{4}{3}\cdot\pi\cdot{R_{h,50}}^{3}} \right)\frac{R_{h,50}}{R_{h}}$$

(eq8)

As one crosslinker connects two subchains, the total number of cross-links is defined as:

$$N_{c}=\frac{1}{2}N$$

(eq9)

The approximation of the total number of sub-chains calculated by: (Balaceanu, A., Demco, D. E., Möller, M. & Pich, A. Heterogeneous morphology of random copolymer microgels as reflected in temperature-induced volume transition and 1H high-resolution transverse relaxation NMR. Macromol. Chem. Phys. 212, 2467–2477 (2011)):

$$N=2\cdot N_{A}\cdot V_{0}\cdot c$$

(eq3)

| $N$ | is the number of sub-chains in a microgel particle |
| --- | --- |
| $N_{A}$ | Avogadro‘s number $6.022140 \cdot{10}^{23} {mol}^{-1}$ |
| $V_{0}$ | $V_{0}=\frac{4}{3}\cdot\pi\cdot{R_{h,50}}^{3}$ |
| $c$ | Molar concentration of cross-linker used in the synthesis process |

Figure 2: pNIPAm microgels with different crosslinker content or size. Hydrodynamic diameter of the microgels calculated from DLS (left) and the calculated shear modulus of microgels (right).

As already mentioned above, the calculation of the shear modulus of the microgels must be judged carefully (Fig. 2, right panel), as several assumptions were made to obtain these values, nevertheless it provides an impression of the range of the microgel shear modulus.

*10. Conclusions made in the discussion, specifically (but not only) "the migration of B16F1 cells is inversely correlated with microgel array spacing" are not supported by the data presented. This needs to be carefully rewritten. Similarly, "In this context, we have developed a (...) cell adhesion and migration". I do not see evidence that the system presented allow to modulate actin cytoskeleton architecture. There is no control in the phenotypes observed and no clear trends when correlating spacing and migration/FA dynamics are observed. The cytoskeleton architecture has not been systematically studied or characterised.*

In our opinion, the statement “the migration of B16F1 cells is inversely correlated with microgel array spacing” is correct and supported by Fig. 9 (with the possible exception of the motility of cells on 1600 nm microgel arrays). As to the statement “In this context, we have developed a …cell adhesion and motility”, we do provide evidence that surface-grafted microgel arrays modulate actin cytoskeleton architecture. Indeed, in Fig. 4, we show that actin cytoskeleton organisation and focal adhesion orientation are phenotypically different on microgel arrays compared to glass. Hence, we can conclude that microgel arrays modulate actin cytoskeleton architecture. Obviously, a systematic characterisation would require the analysis of hundreds of actin-associated proteins, a task which requires more than one study.

*11. The conclusion that "the variation of microgel array topographic and mechanical features can efficiently be used for the modulation of cell adhesion and motility" is misleading. There is no real control achieved (which would be evidenced by trends with microgel size for example). The only trends observed are the response of cells to films and arrays compared to glass. But this could be the result of differences in adhesive protein adsorption promoting differences in cell adhesion.*

In our opinion, the statement "the variation of microgel array topographic and mechanical features can efficiently be used for the modulation of cell adhesion and motility" highlighted by the reviewer is supported by solid experimental evidence. As to the topography, we have not only compared cell motility and adhesion on glass to the same biological processes on films and arrays, but also films to arrays. Furthermore, we have changed arrays spacing (i.e., microgel topography) and clearly show that it can modulate cell adhesion and motility. Also in this case, the comparison was done with glass, but also with films and between pairs of different array spacings (see statistical analysis in the suppl. data). Regarding the mechanical feature (i.e., content of cross-linker), we clearly show that focal adhesion turnover is clearly modulated by this microgel feature in both WT and Gas2L1 KO cells.

*12. Fig. 7. It would be useful to show some of the normalised fluorescence intensities prior to photobleaching in the traces presented.*

As the reviewer certainly knows, the normalised intensities prior to photobleaching are equal to 1 and will be visualised as curves parallel to the X axis for all the conditions (i.e., glass, films and arrays). In our opinion, the information will not add any relevant detail to the figure but introducing it will cause the curves to be squeezed together (because the Y axis will include a range of values up to 1 or more) thus making any difference difficult to appreciate.

*13. The manuscript is sometimes vague and the language used could be more specific. For example, "Focal adhesion dynamics is also modulated by microgels". What properties of microgels? Or, "the kinetics of the focal adhesion protein zyxin is decreased". The kinetics of which phenomenon? Presumably recruitment and disassembly. Similarly, "whereas focal adhesion speed was clearly reduced". This is presumably the FA formation rate, or the FA turnover.*

We have replaced “Focal adhesion dynamics is also modulated by microgel topography”. In the sentence “the kinetics of the focal adhesion protein zyxin is decreased”, the word kinetics clearly refers to the mobility of this protein with focal adhesion. The term kinetics is conventionally used in the field and does not refer to FA disassembly. Likewise, in the sentence “whereas focal adhesion speed was clearly reduced”, the word speed refers to the apparent FA movement relative to the substratum and not to FA turnover or formation rate. This terminology is also conventionally used in the field and we have already defined it the Materials and Methods section on page 11.

**Reviewer #2:**

*Riegert et al investigated how surface bound microgel arrays affect cell adhesion and migration. They controlled and varied the spacing of patterning and degree of crosslinking and showed that cell adhesion and migration are indeed affected. Greater spacing led to increased cell polarization and migration directionality. The author further showed that this is directly correlated with the focal adhesion dynamics. This is a very interesting study, and I find the article generally well written, and conclusions directly supported by the observation. I have a few comments:*

We thank you the reviewer for supporting our study and his/her efforts towards improving it.

*Fig 2 – are the second column a line cross-sectional profile or is it projected vertically? In A, the periodicity is very clear, but seems to be diminished to noise in B. Further, in C, the microgel is much narrower than the surface, but the line profile in D seems to suggest they’re about the same width. The authors should indicate where the cross-section is drawn on the first column that produced the profile on the second column.*

The cross-sections are perpendicular to the line direction. The peaks represent the microgels and the gaps the spacing between them. We have added a white line in each AFM image to indicate where the cross-section was taken and have redrawn the profiles for B, D and F. The small microgels shown in Figure 2 A, B are ~4 nm high and ~200 nm wide when are adsorbed on a surface. Given the range of the Y-axis (0-160 nm), chosen for allowing a direct comparison of the 300, 800, 1200, 1600 nm arrays, it is difficult to see their periodic structure. We have changed the position of the profile, to show more clearly their periodicity. The microgel line in Figure 2 C, D has a width of approximately 450 nm and the line distance is in a comparable range. The difference between C and D is an optical illusion. To avoid this impression, we have redrawn the profile to have a clearer view on the periodicity.

*In the current design, both the gel with and the gap width seems to be increasing at the same time. So, is it the microgel array width or their gap that has the stronger effect on their influence to cell adhesion? For instance, keeping the gels at 300nm width, but spacing hem 1600 nm apart, would the authors expect to see similar result as gel/gap width = 300/300 or 1600/1600?*

The reviewer is right in that the line distance increases with the microgel size at the same time. The PDMS stamp we use has a sinusoidal form, with amplitude and wavelength increasing proportionally. We found that the ratio of the wavelength to the hydrodynamic diameter of the microgel in swollen state must be in the range 1.5-2.5. This limits the printable ratios. If a smaller microgel is printed with a larger stamp the microgels begin to form pyramids. This occurs, in part, in the 1600 nm arrays as the microgel to stamp ratio is at the lower end of the range. Therefore, it is technically not possible to fabricate the geometries suggested by the reviewer.

*What is the mechanism behind the cells’ alignment in the direction of the pattern at 300nm (Fig 8A)? Given the authors stated that the surface swelling made it behave like a connected sheet. How does the cell get the cues to polarize in this context along the patterns. While the authors stated that the molecular mechanism is outside the scope of the current study, can they provide some reasonable interpretations and what they hypothesize might be the cause?*

We thank you the reviewer for highlighting this important point. We do not have an explanation concerning the molecular mechanisms underlying cell alignment on 300 nm microgel arrays. One possible explanation is that even after swelling, 300 nm microgel arrays retain their parallel orientation, which is still and clearly sensed by the cells. In the microgel films, by contrast, the single microgel particle, though very close to each other, are homogenously distributed in all orientations (see, for example, Fig. 4 in Sechi et al. 2016) thus providing no polarization cue to the cells. We have now included this hypothesis in the discussion.

*Fig 9 C, D doesn’t seem as significant as authors stated. For instance, in Fig 9C, film, 800, 1600nm doesn’t seem to be different. Similarly, glass, 300 and 1200 do not seem that different. How many independent experiments did the authors perform, and how was the variation between experiments? Please include statistical test between the necessary groups stated in the manuscript.*

The statistical analysis for Figure 9 is provided in the supplementary Tables S7 and S8. We have analysed 2-3 independent experiments each including at least 100 measurements (for cell motility, FA dynamics, etc.). We now include this information in the main text. As to the variation between experiments, obviously the biological variation introduces fluctuations in the cellular parameters that we have measured (i.e., average speed, focal adhesion dynamics; see figure below for the reviewer). It is important to note that we have analysed and compared cellular parameters at single-cell level and not the average values calculated from single experiments. Moreover, as show in the figure below, the general cellular behaviour and our conclusions remain unaffected regardless of such biological variation (for instance, reduction of average speed as microgel spacing increases in both experiments).

**Reviewer #3:**

*In this work, Sechi and coworkers developed surface-supported microgel arrays featuring different spacing and elasticity and investigated the effect of such topographic and mechanical cues on the adhesion and migration of different cell types. Building up on previous research from their group, the authors optimized the fabrication method of the biomaterial by varying the microgel size and crosslinking degree to control the spacing between adjacent arrays as well as the materials mechanical strength. After characterization of the synthesized microgels and the printed microgel arrays, the authors used this platform to study the response of cells (B16F1 and Sertoli cells) in terms of adhesion and migration in comparison to cells cultured on glass and microgel films. Sertoli cells were found to elongate and align their morphology, actin cytoskeleton and focal adhesions in respect to the orientation of the arrays. Additionally, the rate of motility and directionality were investigated, and a more pronounced impact of microgels on the migration of Gas2L1 KO vs. wild-type Sertoli cells was found, while directionality was not significantly impacted. Microgels topography and its stiffness was as well found as effective means of modulating focal adhesion dynamism. Regarding B16F1 cells, their migration average speed was reduced on microgel topography, and showed dependence on the array spacing (with a lower limit of control found at 300 nm spacing), reflecting differences in focal adhesion dynamics. The findings of this work are useful to understand the influence of different design parameters of microgel culture platforms on cell functionality. In a future perspective, these concepts could guide the development of implantable scaffolds with controlled cell adhesion and migration, to support wound healing and tissue engineering.*

*This is a technically sound investigation: the aim of the work is clearly formulated, the experiments are competently performed, the analysis of different parameters on cell response is exhaustive and the results are well presented. The findings are interesting, the conclusions are supported by the data and the overall quality and clarity of this manuscript are very good. Some experimental details are missing, and some typos need correction, all aspects that can be easily implemented. I positively support this article and recommend the acceptance for publication in PLOS One after minor corrections noted (see below).*

We thank you this reviewer for supporting the publication of our study and the useful remarks.

*1- Experimental Section: some important missing details should be included:*

*- The authors mention the “microgel films” (Page 10) as one of the control substrates to be compared to the “microgel array”. However, the preparation of the microgel films is not described in the Exp. Section. Are they prepared similarly to the microgel arrays, except that the printing part is skipped? Please, complete this part for clarity.*

We have added the missing information in the Materials and Methods section (page 7).

*- Page 6, line 120: “Microgels were subsequently purified by dialysis for several days”. How many days were needed typically? Please, inform average purification time.*

We have included the missing information.

*- Page 8, formula to calculate the degree of microgel swelling: Please, define the different terms or magnitudes of the equation at first mention. In connection to that, how were the swelling degree and the volume phase transition temperature measured? I suggest mentioning it briefly, even when they are shown in the Supp. Info.*

We have changed the text as suggested by the reviewer.

*- Page 25, legend of Fig. 7, lines 554-555: “The thin lines above and below the thick curves indicate the S.E.M.” Please, define “S.E.M”.*

We have now defined S.E.M. (standard error of the mean).

*2- There are some typos to correct in the text:*

*Page 4, line 90: “regulate” instead of “regulated” - The authors vary between “cross linking” and “crosslinking” throughout the text. Please, unify style. Same for “cross linker”, “crosslinker” and “cross-linker”.- Supporting Information: “FTIR spectra” instead of “FITR spectra”.- Page 25, line 565: “chose” instead of “chosen”.- Page 31, line 715: “ratio” instead of “ration”.*

We have corrected these typing errors.

*3- My last comment is more a suggestion to the authors. With the investigation of design parameters (spacing and stiffness) on diverse cell responses (morphology, cytoskeleton and focal adhesion alignment, focal adhesion turnover, migration rate and directionality, etc), across different substrates (glass, microgel array, microgel films) and cell types (B16F1 cells, and wild-type vs Gas2L1 KO Sertoli cells), at some point the reader may get lost with so many data. I believe that a clear summary of the different findings at the end of the discussion section will be very appreciated by the reader and will nicely wrap up the main findings. This could be implemented, for example, in the form of a table that compares which factor has a significant influence on a given response.*

We thank the reviewer for the suggestion. We now include two new tables (Table 2 and 3), which summarise the main observations of our study.
